# Societal Learning in Epidemics: Intervention Effectiveness during the 2003 SARS Outbreak in Singapore

**DOI:** 10.1371/journal.pone.0000020

**Published:** 2006-12-20

**Authors:** John M. Drake, Suok Kai Chew, Stefan Ma

**Affiliations:** 1 National Center for Ecological Analysis and Synthesis Santa Barbara, California, United States of America; 2 Ministry of Health Singapore, Singapore; James Cook University, Australia

## Abstract

**Background:**

Rapid response to outbreaks of emerging infectious diseases is impeded by uncertain diagnoses and delayed communication. Understanding the effect of inefficient response is a potentially important contribution of epidemic theory. To develop this understanding we studied societal learning during emerging outbreaks wherein patient removal accelerates as information is gathered and disseminated.

**Methods and Findings:**

We developed an extension of a standard outbreak model, the simple stochastic epidemic, which accounts for societal learning. We obtained expressions for the expected outbreak size and the distribution of epidemic duration. We found that rapid learning noticeably affects the final outbreak size even when learning exhibits diminishing returns (relaxation). As an example, we estimated the learning rate for the 2003 outbreak of severe acute respiratory syndrome (SARS) in Singapore. Evidence for relaxation during the first eight weeks of the outbreak was inconclusive. We estimated that if societal learning had occurred at half the actual rate, the expected final size of the outbreak would have reached nearly 800 cases, more than three times the observed number of infections. By contrast, the expected outbreak size for societal learning twice as effective was 116 cases.

**Conclusion:**

These results show that the rate of societal learning can greatly affect the final size of disease outbreaks, justifying investment in early warning systems and attentiveness to disease outbreak by both government authorities and the public. We submit that the burden of emerging infections, including the risk of a global pandemic, could be efficiently reduced by improving procedures for rapid detection of outbreaks, alerting public health officials, and aggressively educating the public at the start of an outbreak.

## Introduction

Rapidly spreading outbreaks of infectious diseases are an increasing concern for global public health [1], [2] and security [3]. Emerging infections, which are typically defined as infectious diseases that have newly appeared in a population or are rapidly increasing in incidence or geographic range [4], are a particular concern because at the time of emergence little is known about their epidemiology, particularly pathology, symptomatology, and transmissibility. Thus, the crucial tasks of assessing epidemic risk and determining what public health interventions should be taken are complicated by uncertainty that borders on complete ignorance. Of course, this uncertainty is rapidly reduced as the outbreak progresses and information concerning symptoms of infection, the biology of the infectious agent, the epidemiology of transmission, and the effectiveness of health precautions and intervention is collected and disseminated.

This learning process has not been considered in theories of outbreak control [5], [6] or in near real-time models of emerging infections [7], [8] (compare correspondence in refs [9], [10]). Here, we study the collective effects of various processes (including possibly unidentified phenomena) on the change in the rate at which infectious persons are isolated. We refer to this set of processes collectively as “societal learning”. A partial list of the processes contributing to societal learning includes isolation and identification of the infectious agent, development of tests for clinical diagnosis, disseminating information to public health and medical personnel, disseminating information to the public, and implementing public health policies including restrictions on individual movement or quarantine.

Disease control theory focuses on an quantity called the reproductive ratio, designated here as *R*_0_ at the start of the outbreak and, if changing over time, *R_t_* at time *t*. Outbreaks are considered to be under control when *R_t_*<1, implying that outbreak conditions are such that on average disease prevalence will decline. Most research in theoretical epidemiology has focused on how *R_t_* is related to disease and population parameters in order to understand how to induce the change from *R*_0_>1, during emergence, to *R_t_*<1. Recent developments include techniques for estimating *R*_0_ from the initial stages of an outbreak [11], [12] and a model to ascertain the effect of a delay between the onset of an outbreak and the implementation of public health policies aimed at controlling disease spread [13]. Here, we contribute to this developing toolbox for disease forecasting a model to understand how societal learning affects the expected final size and duration of disease outbreak. Though some computational disease-specific models have recognized the importance of time-varying rates in disease spread, particularly with respect to the outbreak of SARS in 2003 [14], [15] (compare [16]), we believe this is the first analytical treatment of the concept.

We also retrospectively explore the effect of societal learning during the 2003 outbreak of SARS in Singapore, using weekly data on the time between onset of symptoms and removal of infectious individuals. We speculate that societal learning will generally exhibit diminishing returns because increasing the removal rate becomes more difficult as individual isolation approaches a theoretical maximum rate. In such a case, the rate of societal learning is said to relax. We introduce statistical models to distinguish between relaxing and non-relaxing learning and test for relaxation during this outbreak. Finally, we discuss societal and epidemiological factors that might affect societal learning, we observe that a difficult task during the early stages of an outbreak is to estimate the learning rate and suggest that the rate estimated here might be used as prior information in future outbreaks, and we conclude by recommending rapid investment in research at the time of initial detection when actions taken to reduce disease spread can be most efficient and cost effective.

Public health officials routinely make judgments whether or not to raise alarms about developing outbreaks. This decision is complicated by severe uncertainty during the early phases of an outbreak. Further, bureaucratic inertia and the ignorance that necessarily accompanies emerging infections discourage rapid response. By contrast, false alarms resulting from hasty and premature assessment of outbreak risk can be very costly, and must be avoided if possible. Understanding the role of societal learning in disease outbreaks is important for properly balancing these competing objectives.

## Methods

### Basic theoretical model

Our concept of societal learning is characteristically reflected in outbreak dynamics as an increase over time in the rate at which infectious individuals are removed from circulating in the population. That is, we expect that as information about clinical symptoms, modes of transmission, the duration of incubation, *etc.*, is collected and disseminated, the average time between the onset of symptoms and individual self-removal from the population (for instance by admission to hospital) or forced isolation (*e.g.*, quarantine) will decline. From a dynamical perspective, we represent the average removal rate of individual cases as a function of time since the outbreak began, marked by the time at which the index case became infectious. For emerging diseases we assume that direct transmission between infected persons is the primary source of infection and that development of immunity and removing infectious individuals have negligible impact on the susceptible population. These assumptions are reasonable for outbreaks that ultimately do not infect more than a small fraction of the total population, *i.e.*, emerging infections with relatively low prevalence. Finally, we assume that transmission is a Markov process, an approximation that amounts to assuming that individual infectious contacts are independent (compare [17]). Thus, representing the individual rate of infection by the constant parameter β_0_ and the rate of removal as a function of time γ(*t*), these assumptions imply that the growth of the epidemic is a time-inhomogeneous stochastic birth-death chain [18]–[20]. Accordingly, the change over time in the probability distribution of the number of infected individuals *x* is given by
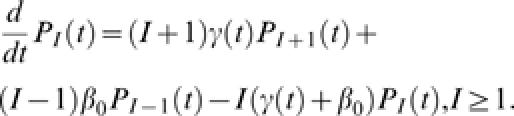



This model has been previously studied and applied to problems ranging from population dynamics to astronomy [18], [19], [21]. In particular, the expected final epidemic size for this model is [18]:
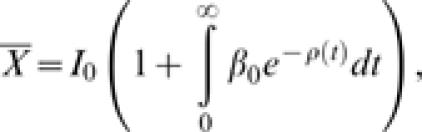
where
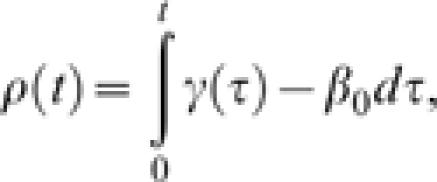
and *I*_0_ is the initial number of infected individuals. Further, the distribution function for the duration of the outbreak with *I*_0_ = 1 is:
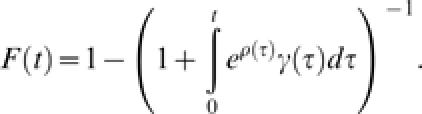



This is a very general model, as we have only specified that the transmission rate β_0_ is constant and that the rate of removal γ(*t*) changes over time, consistent with the concept of societal learning.

### Societal learning

**Figure 1:**
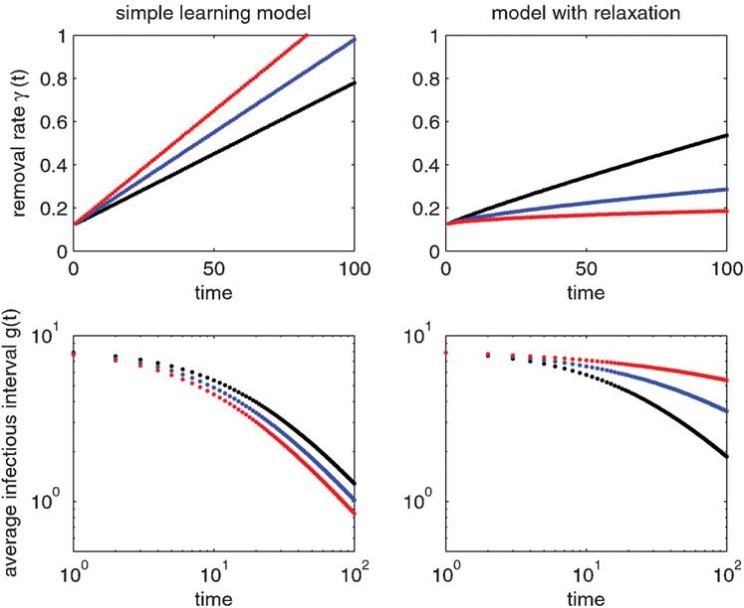
Examples showing the effect of societal learning on removal rate (γ) and average duration between infection and removal (*g*). Plots on the left are for the simple model with no relaxation. Compare with plots on the right ranging from modest to severe relaxation. Denoting the vector of parameters ***ψ*** = [*a*_0_, *a*_1_, *b*], plots on the left are for ***ψ*** = [0.0066, 1, 0.12] (black), ***ψ*** = [0.0086, 1, 0.12] (blue), ***ψ*** = [0.0106, 1, 0.12] (red). Plots on the right are for ***ψ*** = [0.0066, 0.9, 0.12] (black), ***ψ*** = [0.0066, 0.7, 0.12] (blue), ***ψ*** = [0.0066, 0.5, 0.12] (red). The units in which time is measured do not affect the form of these plots. However, for comparison the x-axis can be interpreted in units of days in which case the black points on the left side coincide with the parameter estimates reported in Table 1. Axes on plots of *g* are log-log.

Conceptually, we decompose the removal rate, γ(*t*), into two components. The first component represents removal in the absence of societal learning (*i.e.*, through unexceptional health procedures or natural recovery) and is referred to as the base removal rate. The second component is an effect of societal learning and is assumed to be additive to the base removal rate. Consequently, we represent the total removal rate as function of time γ(*t*) = *a*(*t*)+*b* where *b* is the base removal rate and *a*(*t*) is a function for the additional effect of societal learning. (Refer to Table 1 for biological interpretations of parameters discussed in this section.) Next, we consider two different learning scenarios. First, we suppose that societal learning is constant, *i.e.*, that over any interval a doubling in time since the outbreak began corresponds to a doubling in the learned component of the removal rate. Then, the effect due to learning can be represented as a line *a*(*t*) = *a*_0_ *t*, where *a*_0_ is called the ‘basic learning rate’, and the removal rate is linear: γ_1_(*t*) = *a*_0_ *t*+*b*. Special cases of this model have *a*_0_ = 0, where there is no effect of societal learning (resulting in the simple stochastic epidemic), and *b* = 0 where there is no natural recovery. The model with linear removal rate implies that the average time between infection and removal over time follows a hyperbola, *g*(*t*) = (*γ*_1_(*t*))^−1^ = (*a*_0_ *t*+*b*)^−1^, and that there is no upper bound to the rate at which infected individuals can be isolated; effectively, we suppose that the average time between infection and removal can be brought arbitrarily close to 0. For most (perhaps all) diseases this is an unreasonable assumption in the long run (though it may be a reasonable approximation at the start of an outbreak). In particular, the effect of societal learning probably decreases as the removal rate gets high and the interval between the onset of symptoms and isolation approaches a minimum biologically plausible quantity. This is a scenario in which cumulative number of removed patients is a decelerating function of time marked by diminishing returns. To incorporate such relaxation in our model we should generalize *a*(*t*) for instance *a*(*t*) = *a*_0_ *t^a1^*, with *a*_1_≤1. Where *a*_1_ = 1 this model is equivalent to the linear model discussed above. Of course, there is no principled theoretical reason why *a*_1_ cannot be greater than 1. Such a case is unlikely, however, and would imply acceleration not only in removals, but in the removal rate. In either case we have the general model for the removal rate *γ*_2_(*t*) = *a*_0_ *t^a1^*+*b* and the associated model of the duration of the interval between onset of symptoms and removal *g*_2_(*t*) = *γ*_2_(*t*) = (*a*_0_ *t^a1^*+*b*)^−1^. In this case *g*(*t*) is approximately a power law with respect to time. We remark that learning relaxation could also result from diminishing returns on methods for disseminating information. For instance, if diagnostic information is transmitted by word-of-mouth, models for the spread of a rumor suggest that the fraction of the population which remains uninformed declines roughly logistically: first approximately proportional to the number of people who are in possession of the rumor but declining constantly over time as uninformed individuals become increasingly rare [6], [22]. Examples of γ_1_ and γ_2_ and the associated *g*_1_ and *g_2_* are shown in Figure 1.

**Table 1:**
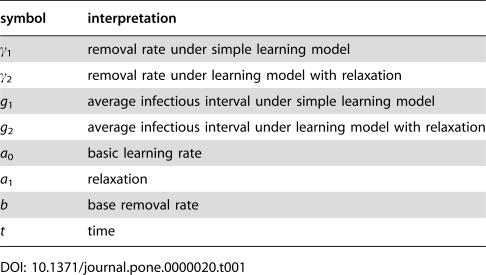
Interpretation of parameters related to societal learning.

symbol	interpretation
γ_1_	removal rate under simple learning model
γ_2_	removal rate under learning model with relaxation
*g*_1_	average infectious interval under simple learning model
*g*_2_	average infectious interval under learning model with relaxation
*a*_0_	basic learning rate
*a*_1_	relaxation
*b*	base removal rate
*t*	time

Substituting the above model for societal learning in eqns (2) and (4) obtains two quantities of special interest: the expected outbreak size,
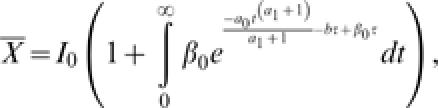
and the distribution of extinction times,
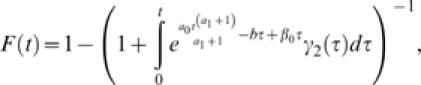
from which the probability density of the duration of outbreaks is obtained as the derivative with respect to time,
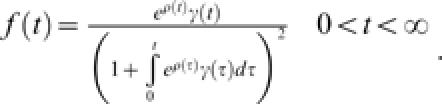



Finally, in this representation of the epidemic process, the concept of the reproductive ratio (designated by *R*_0_ at the beginning of the outbreak, *R_t_* thereafter) is deterministic and is given by *R_t_* = *β*_0_/*γ*(*t*). Setting this equation to one and solving for *t* obtains the time until the outbreak is brought under control. For the case γ(*t*) = γ_1_(*t*), the time to control is given by *T_c_* = (*β*_0_−*b*)/*a*_0_.Still more models could be considered. However, we report below that the final epidemic size is affected mostly by the parameter *a*_0_, the rate of societal learning at the beginning of the outbreak, so that the precise shape of the removal function does not greatly matter.

### Data and test for societal learning

To test for societal learning in the 2003 outbreak of SARS in Singapore, we used the mean number of days between the onset of clinical symptoms and removal, by week, to fit different models for the removal process γ. These data are slightly different than those that appeared previously as Figure 1 in [15] and include some reclassified cases based on serological tests (S. Ma, unpublished data). Societal learning models were fit to the reciprocal of the mean of observed lags between onset of symptoms and removal *γ*_i_ = 1/*g*_i_ for each week *i*, using nonlinear least squares regression. Model fit was assessed using Akaike's Information Criterion (AIC) assuming the observations are drawn from a normal distribution with mean γ*_i_* and homogeneous variance. We tested three hypotheses: (i) the null hypothesis of no base removal rate corresponding to *b* = 0; (ii) the null hypothesis of no saturation in learning corresponding to *a*_1_ = 1; finally, (iii) the null hypothesis of no societal learning at all is given by *a*_1_ = 1 for *a*_0_ = 0.

### Simulation

**Figure 2:**
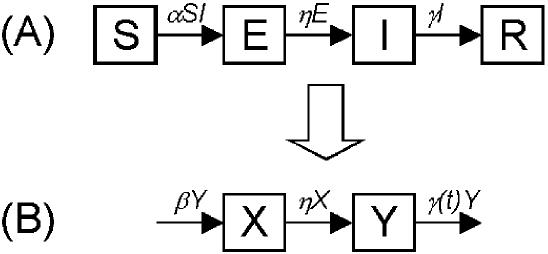
(A) Basic S-E-I-R compartmental model of infectious disease, in which outbreak dynamics are represented by the number of individuals in four compartments corresponding to susceptible, exposed, infectious, and removed (or recovered) individuals. The rate at which individuals move from susceptible to exposed is according to mass-action dynamics with proportionality constant *α*. Individuals move from exposed to infectious at rate *η* and from infectious to removed at rate *γ*. (B) By assuming that the number of susceptible individuals is approximately constant (an appropriate approximation for outbreaks in which prevalence is never a large fraction of the total population) we introduce the new variable β = α*S* and reduce the four-compartment S-E-I-R model to a two-compartment model, designated here by the state variables *X* and *Y*.

To represent the full epidemic process for SARS the societal learning theory developed above must be modified to account for a significant latent period [12]. Accordingly, we adopt the familiar S-E-I-R modeling framework (Figure 2A in [5]), modified to represent stochastic (Markov) dynamics with time-inhomogeneous parameters. As before, we adopt the reasonable assumption that the population is large compared to the eventual size of the outbreak so that *S* remains constant throughout. Thus, by substituting β_0_ = α*S* and ignoring the dynamics of removed individuals, we obtain the two-compartment model in Figure 2B, where *X* and *Y* designate the classes that were formerly *E* and *I*. Finally, consistent with our earlier definition of societal learning, we allow the removal rate γ to be a function of time, designated γ(*t*). We assume that each state variable *X* and *Y* can take only integer values (demographic stochasticity) and that individual transitions between classes are Markovian. This model is a pair of coupled birth-death chains and is a generalization of the model studied in the earlier part of this paper.

We obtained parameter values for these simulations as follows. Using a Bayesian approach, Lipsitch et al. [15] determined that the basic reproductive ratio (*R*_0_) for this outbreak was in the range [2.2, 3.6]. These values accord well with the likelihood-based estimate of Wallinga and Teunis [23], who report a point estimate of *R̂*_0_ = 3.1 and 95% confidence interval [2.3, 4.0]. Interpreting the estimates of Lipsitch et al. [15] as the rate of secondary infection in a wholly susceptible population, *R*_0_ is related to our parameters through the relation β_0_ = *R*_0_×γ_0_. Recognizing that uncertainty in both *R*_0_ and γ will affect the accuracy of model projections we obtain an upper limit on β_0_ (not a confidence interval because the parameters are not independent) from β^+^ = *R*_0_^+^×γ^+^ and a lower limit from β_0_^−^ = *R*_0_^−^×γ^−^, where (+) and (−) indicate the upper and lower limits on the estimate intervals for the respective parameters. To obtain a central (“best”) estimate of β_0_ we take the midpoint of the range [2.2, 3.6] = 2.9 and multiply by the point estimate of our regression *γ̂*_0_ = 0.12 to obtain *β̂* = 0.35. Throughout, we used the point estimate from the regression analysis above (0.046, see also *Results*) for the basic learning rate after dividing by seven to convert from weeks to days: *a*_0_ = 0.0066. As the learning rate never declined over the course of this outbreak, no relaxation was included in the model. Finally, the transition rate between latent and infectious individuals (η) is approximately equal to the reciprocal of the duration of the incubation period. We used a transition rate of 0.15 d^−1^, corresponding to an average incubation period of approximately 6.7. days. This is roughly consistent with, *e.g.*, the ranges of estimates compiled by the World Health Organization (Table 1 in [24]) and the estimate (6.37 d) and 95% confidence interval [5.29, 7.75] reported by Donnelly et al. [25], but slightly larger than the estimate of 4.8 d (95% confidence interval: [4.37, 5.29]) obtained by Kuk and Ma [26] under the assumption that incubation times are drawn from a Weibull distribution.

### Comparison between model predictions and observed outbreak size

Retrospectively comparing model-based estimates of the expected outbreak size with the 238 observed cases (a partially circular comparison to begin with) is complicated by the fact that the number of initially infected individuals (the initial condition) is not defined by the model but must be asserted. One possibility is to assume that the outbreak begins with the index patient (*I*_0_ = 1), but then the outbreak size of the theoretical model is biased by a significant portion of outbreaks that fail due to stochastic fadeout [27]. An alternative is to compare the observed outbreak size with the theoretical distribution of outbreak sizes for outbreaks initialized at *I*_0_ = 1 conditioned on a ‘major’ or ‘observable’ outbreak occurring. However, this simply pushes back the problem of specifying the initial condition as some number of cases must be specified to correspond with ‘major’ or ‘observable’. We adopted a third alternative. We reasoned that the first time medical personnel are alerted to the fact that there might be an emerging outbreak is the time that the index patient is observed to be infectious, corresponding to the removal of the patient from the population. At this time, the patient has infected an expected additional *R*_0_ individuals (by the definition of *R*_0_) and these infectious, or soon-to-be-infectious individuals are circulating in the susceptible population. We refer to this as the ‘second generation initialization’. Alternatively, the hospitalization of one individual with an anomalous infection is unlikely to attract significant attention. Consideration of a possible outbreak more likely corresponds to the admittance in quick succession of several patients with anomalous infections, that is when the second generation of infected individuals is isolated and a third generation of individuals is infected. This is the ‘third generation initialization’. Accordingly, we simulated two distributions of final outbreak sizes. First we initialized at *I*_0_ = 3, which is the midpoint of the estimated interval for *R*_0_ identified by Lipsitch et al. [15], *I*_2_ = *R̂*_0_ = 2.9, rounded to the nearest integer, corresponding to second generation initialization. Second, we initialized at *I*_0_ = 8, which is the rounded value of the expected number of infected individuals in the third generation, *I*_3_ = *R̂*_0_^2^ = 8.4. To understand the importance of societal learning during the actual outbreak in Singapore, we simulated 10,000 iterations of the stochastic S-E-I-R model described above using Gillespie's direct method [28] with double and half the estimated basic learning rate while all other parameters were set to their best estimates and with initial condition *I*_0_ = 8. Empirical quantiles and the coefficient of variation (a measure of dispersion, the ratio of the standard deviation to the mean) were used to summarize the distributional properties of simulations.

## Results

### Effects of societal learning on final epidemic size

**Figure 3:**
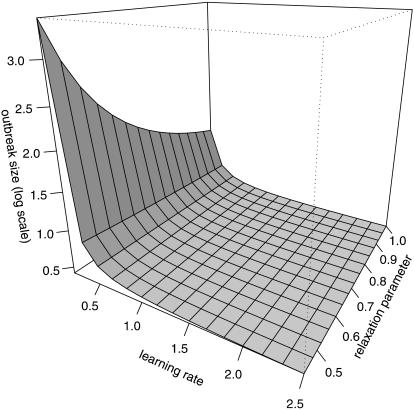
Effects of societal learning and learning relaxation on the expected outbreak size in a stochastic epidemic model.

To look at the effects of societal learning and relaxation on outbreak control, we studied the average outbreak size over a range of scenarios (Figure 3). For simplicity, we assumed β_0_ = 1 throughout and compared different versions of the removal and learning process by tuning the parameters for the basic learning rate (*a*_0_) and the relaxation rate (*a*_1_). The temporal resolution of this model is therefore not explicit. Thus, for concreteness assume that all rates are in units of days and that the baseline infectious period (*g* = γ^−1^) is 3 d. Then, the basic reproductive ratio is *R*_0_ = *β*_0_/γ = 3 and we obtained the average epidemic size from eqn (5) for combinations of *a*_0_ and *a*_1_ in the ranges 
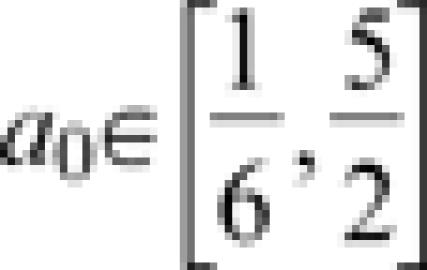
 and *a*_0_∈[0.44,1]. These ranges illustrate the range of cases between extremes in which societal learning is slow and relaxation is rapid (practically no effect of societal learning) and where societal learning is fast and no relaxation occurs at all (similar to the outbreak of SARS). Figure 3 shows that *a*_0_, the basic rate of societal learning, can be important for controlling outbreaks. The effect of relaxation can be examined by comparing the average outbreak size at various values of *a*_1_<1 with the value at *a*_1_ = 1, where there is no relaxation. Evidently, relaxation must be extremely rapid (around *a*_1_ = 0.5) for the effect to be noticeable. Of course, this phenomenon is accentuated by its interaction with the basic societal learning rate so that if learning is extremely slow the effect of relaxation becomes more important.

### Societal learning during the 2003 SARS outbreak in Singapore

**Figure 4:**
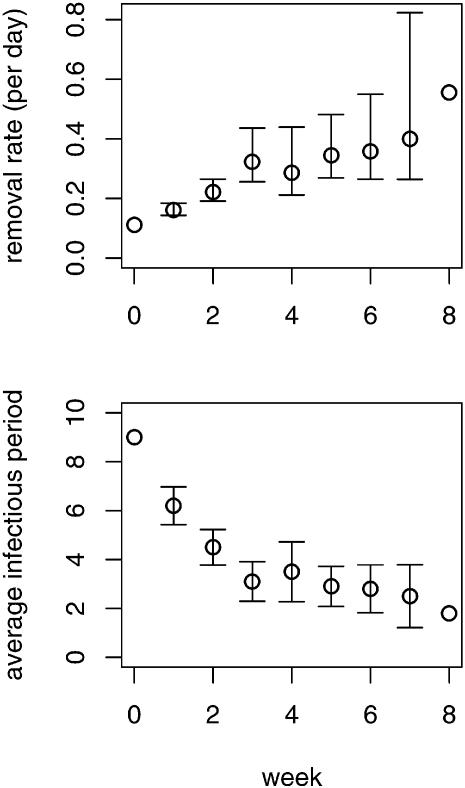
Average daily removal rate of infectious individuals (γ) increased consistently for eight weeks following the initial outbreak of SARS in Singapore in 2003. Average infectious period obtained as *g* = 1/γ. Error bars are 95% confidence intervals calculated from the t-distribution given the mean and standard deviation of observed intervals between onset of clinical symptoms and removal. Confidence intervals are not provided for week 0, where only one case was observed (so zero degrees of freedom), or week 8, where the combination of high standard deviation in the observed interval (s.d.: 1.9) and few degrees of freedom (d.f.: 5) results in a nonsensical confidence interval that includes zero and negative values.

The observed removal rate increased consistently over the course of the 2003 SARS outbreak in Singapore (Figure 4). We found no effect of relaxation in the rate of societal learning, although there was strong evidence for both a baseline removal rate and an effect of learning (Table 2). We first fit the full model, but failed to reject the null hypothesis of no relaxation. Consequently, we fit the reduced model with a constant learning rate, which is equivalent to the full model with exponential parameter *a*_1_ = 1. In this model, both the base removal and learning parameters were highly significantly different than zero (base: *P* = 0.002; learning rate: *P*<0.0001). We remark that the reciprocal of the estimated base removal rate (*b*) can be interpreted as the duration of the infectious period in the absence of special intervention. Accordingly, we obtained an estimate of 8.3 d (95% confidence interval: [5.8, 14.3], obtained by inverting the confidence limits reported in Table 2).

**Table 2:**
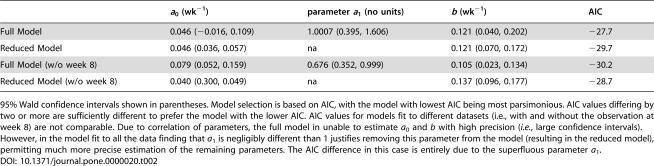
Parameter estimates for a model of societal learning in the 2003 SARS outbreak in Singapore.

	*a*_0_ (wk^−1^)	parameter *a*_1_ (no units)	*b* (wk^−1^)	AIC
Full Model	0.046 (−0.016, 0.109)	1.0007 (0.395, 1.606)	0.121 (0.040, 0.202)	−27.7
Reduced Model	0.046 (0.036, 0.057)	na	0.121 (0.070, 0.172)	−29.7
Full Model (w/o week 8)	0.079 (0.052, 0.159)	0.676 (0.352, 0.999)	0.105 (0.023, 0.134)	−30.2
Reduced Model (w/o week 8)	0.040 (0.300, 0.049)	na	0.137 (0.096, 0.177)	−28.7

95% Wald confidence intervals shown in parentheses. Model selection is based on AIC, with the model with lowest AIC being most parsimonious. AIC values differing by two or more are sufficiently different to prefer the model with the lower AIC. AIC values for models fit to different datasets (i.e., with and without the observation at week 8) are not comparable. Due to correlation of parameters, the full model in unable to estimate *a*_0_ and *b* with high precision (*i.e.*, large confidence intervals). However, in the model fit to all the data finding that *a*_1_ is negligibly different than 1 justifies removing this parameter from the model (resulting in the reduced model), permitting much more precise estimation of the remaining parameters. The AIC difference in this case is entirely due to the superfluous parameter *a*_1_.

**Figure 5:**
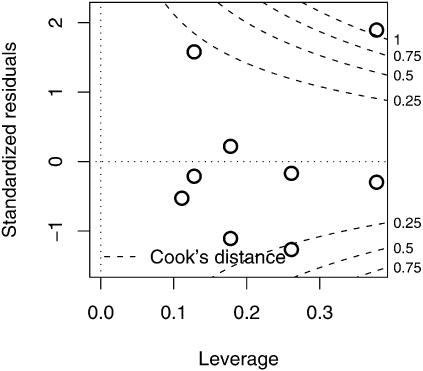
Plot of standardized residuals vs. leverage for nine observations used in the statistical model. One point (corresponding to week 8, in the upper right hand corner of the plot) exhibits high leverage and falls outside the Cook's distance contour at *C* = 1. As this point may have unduly influenced the estimated model, the full and reduced models were re-fit to the data excluding this point.

Inspection of the plots in Figure 4 suggests that the observation in week 8 may be of exceptional importance to the final model. In terms of regression diagnostics, it may have high leverage (greatly affecting the uncertainty in parameter estimates) and high influence (greatly affecting the estimates themselves). A plot of standardized residuals versus leverage for the reduced model shows that this point is indeed matched by only one other point (week 0) for leverage (Figure 5). Overlaying contour intervals for Cook's distance, a measure of influence, shows that this point also has high influence. Accordingly, so that the reader may compare we re-fit both the full and reduced models after dropping this point (Table 2). In this case the AIC difference is less than two, so that neither model is better supported by the data. Further while the maximum likelihood estimate for *a*_1_ is quite low (*a*_1_ = 0.676; to be interpreted as considerable relaxation), the confidence interval barely fails to include 1, so the evidence is not conclusive.

### Effect of latent period

**Figure 6:**
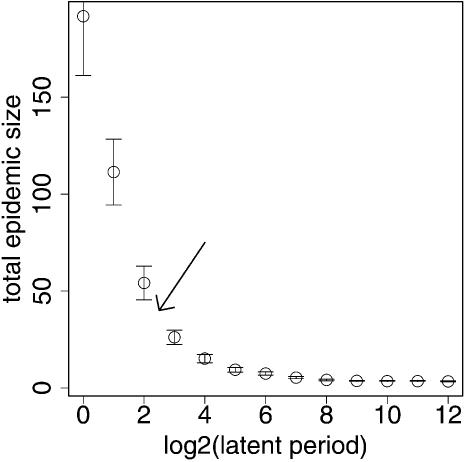
Relationship between the average latent period (x-axis) and average total outbreak size in simulations (y-axis). Latent period is log_2_ transformed (to illustrate a wide range of possible values) and ranges from 1 d to 4096 d (∼11 y). The approximate location of SARS is indicated by the arrow.

To study the effect of the duration of the latent period on average outbreak size, we simulated 500 iterations of the model at each of 13 different durations for the average latent period. The average outbreak size decreased with the duration of the latent period as shown in Figure 6.

### Comparison between model and observed outbreak size

The average size of simulated outbreaks initiated with the second generation initialization condition (*I*_0_ = 3) was 102 cases. The 2.5% and 97.5% quantiles were 4 and 321 cases, respectively. The coefficient of variation in the final outbreak size was 0.85. The average size of simulated outbreaks initiated with the third generation initialization condition (*I*_0_ = 8) was 278 cases. The 2.5% and 97.5% quantiles were 56 and 611 cases, respectively, with coefficient of variation 0.52. Thus, the observed total outbreak size (238 cases; [29]) is consistent with either the second or the third generation initialization conditions. Outbreak simulations in which learning occurred at half the observed rate had average final outbreak size of 799 cases while outbreak simulations in which learning occurred at twice the observed rate had average final outbreak size of 116 cases.

## Discussion

We found little evidence for relaxation in the learning rate for SARS in Singapore. First, restricting our discussion to the analysis with all data, we find that the maximum likelihood estimate of the relaxation parameter is extraordinarily close to one (differing by 0.07%), perfect non-relaxation. Admittedly, the confidence interval on this parameter is large. One interprets this to mean that the vigilance of the public health community as a whole continued throughout the outbreak and that improvement in intervention effectiveness continued unabated. However, we also found that one relatively uncertain data point was important to this analysis (week 8). Whether this point should be excluded from interpretation is unclear. On one hand, it is a real observation and (because of its high influence) is known to contain a great deal of information. Therefore, one is inclined to allow this observation considerable weight. On the other hand, its importance, especially at the end of the data series is suspicious. If we exclude this point from analysis *post hoc*, we find that we are unable to make any strong conclusions at all. What most likely occurred is that the distribution of average infectious period at the point where the outbreak was rapidly brought under control was highly dispersed (high variance) and highly skewed. Accordingly, the mean removal rate probably does relax, but the data that were available to this study are too aggregated to make this inference conclusively.

It is unknown if the rate of learning estimated in this study is unique to this outbreak or if it might be more representative. We remark that both parameters in the learning rate model are readily interpreted, and that theoretical effects of improvement in surveillance, mechanisms for informing public health personnel and the public, and rapid research response could be studied by extending this simple model to represent more realistically the effects of alternative policies as covariates.

The final size of an outbreak is greatly affected by transmission events early during the outbreak process. Outbreaks can be curtailed when public health interventions are rapid and efficient. But the severity of an outbreak is often unclear during these initial stages of transmission when intervention can be most effective [11], [27]. Further, there are limits to how quickly diagnostic information about an emerging infection can be obtained and disseminated to health care providers. This is not the first model to consider the effect of changes in the removal rate (*e.g.*, [14], [15]). However, in contrast to earlier studies, we first explicitly considered societal learning parametrically in a theoretical model. Our model also more realistically represents the ramping up of intervention in contrast to models that simply have “before control” and “after control” regimes (*e.g.*, [13]). We showed that the final outbreak size decreases rapidly with a modest investment in learning. We also found strong evidence of learning in data from the 2003 outbreak of SARS in Singapore. Public health interventions for SARS include encouragement to report to hospital rapidly after the onset of clinical symptoms, contact tracing for confirmed and suspected cases, and quarantine, monitoring, and restricting the travel of contacts [25], [30]. We believe these interventions were highly effective at reducing the final size of the SARS outbreak.

A limitation of this analysis is that we only consider temporal changes in removal, though information dissemination and public concern almost certainly led to a decline in transmission (β_0_) too [15]. Unfortunately, this effect is much more difficult to independently estimate and must instead be inferred from the information provided by the epidemic curve together with observations of the onset-of-symptoms to removal interval. In general, however, the model studied here (eqn 1) and its solution (eqn 2) will also apply to this situation and can be used wherever such data are available. The effects of biological and social factors that might bring about changes in transmissibility is an important area for further theoretical research.

Our estimate of the duration of the infectious period (8.3 d, 95% CI: [5.8, 14.3]) is consistent with measures of viral shedding, obtained by Peiris et al. [31] using quantitative reverse transcriptase on sequential nasopharyngeal aspirates/throat and nose swabs (NPA/TNS), in which maximum virus excretion occurs around the tenth day of illness (compare also [24]). Indeed, only about 35% of NPA/TNS continued to test positive by the third week since the onset of symptoms [24].

These results underscore the value of immediate action at the start of an outbreak (high *a*_0_). The processes considered to contribute to societal learning include such publicly visible actions as declaring a state of emergency, global health alert, or (minimally) disseminating information to the public. The societal and economic costs of mistakenly declaring a state of emergency can be tremendous, but are probably small in comparison to the costs of failing to intervene in a major preventable outbreak. Thus, we echo Anderson et al. [32] in concluding that the major lessons of the 2003 outbreak of SARS are to improve surveillance and detection, including real-time data collection; develop capability for rapid response by the research community; and devise mechanisms for immediate implementation of effective interventions. Important topics for research include estimating the effect of learning on transmission (the parameter β_0_ in the model), and identifying the different activities that contribute to learning (*a*_0_) and relaxation (*a*_1_) and their costs. Then, a cost sensitive model should be developed to balance the competing goals of raising unnecessary alarm and preventing a major outbreak. Such a model would be most useful if it had reference points that would trigger alerts at different levels (*i.e.*, to function as an early warning system) and could guide intervention efforts. Such a model would not need to be purely economical, but could incorporate loss of human life and well-being as constraints on the decision set.

Of course, learning rates (and possibly relaxation) will vary geographically reflecting different societal conditions, research institutions, levels of emergency preparedness, *etc*. Further, these phenomena may also differ among emerging diseases, for instance depending on their similarity to diseases that are well understood or their resistance to laboratory isolation and characterization. Despite these limitations, we suggest that our estimate of the basic learning rate (0.0066 d^−1^; 95% confidence interval [0.0051, 0.0081]) could be used as prior information during future outbreaks. The difficulty of forecasting the total epidemic curve at an early stage is well appreciated [7]. By eliminating the need to simultaneously estimate highly correlated parameters, a good understanding of the dynamical consequences of public health response would enable real-time modeling to focus on estimating disease parameters like transmission rates [11]. Then, estimated disease components and known or conjectured models for response, including models of societal learning, could be integrated in a single modeling framework for projections.
